# Atomic Scale Mechanisms Controlling the Oxidation of Polyethylene: A First Principles Study

**DOI:** 10.3390/polym13132143

**Published:** 2021-06-29

**Authors:** Yunho Ahn, Xavier Colin, Guido Roma

**Affiliations:** 1 Université Paris-Saclay, CEA, Service de Recherches de Métallurgie Physique, 91191 Gif sur Yvette, France; yunho.ahn@cea.fr; 2 PIMM, Arts et Metiers Institute of Technology, CNRS, CNAM, HESAM University, 151 Boulevard de L’Hôpital, 75013 Paris, France; xavier.colin@ensam.eu

**Keywords:** polyethylene, thermal oxidation, radio-oxidation, density functional theory, hydroperoxides, chemical kinetics

## Abstract

Understanding the degradation mechanisms of aliphatic polymers by thermal oxidation and radio-oxidation is very important in order to assess their lifetime in a variety of industrial applications. We focus here on polyethylene as a prototypical aliphatic polymer. Kinetic models describing the time evolution of the concentration of chain defects and radicals species in the material identify a relevant step in the formation and subsequent decomposition of transient hydroperoxides species, finally leading to carbonyl defects, in particular ketones. In this paper, we first summarize the most relevant mechanistic paths proposed in the literature for hydroperoxide formation and decomposition and, second, revisit them using first principles calculations based on Density Functional Theory (DFT). Our results partially confirm commonly accepted reaction energies, but also propose alternative, more favourable, reaction paths. We highlight the influence of the environment—crystalline or not—on the outcome of some of the studied chemical reactions. A remarkable result of our calculations is that hydroxyl radicals play an important role in the decomposition of hydroperoxides. Based on our findings, it should be possible to improve the set of equations and parameters used in current kinetic simulations of polyethylene radio-oxidation.

## 1. Introduction

Polyethylene (PE) is one of the most common polymers and is employed in a variety of applications such as common packaging plastic, domestic sectors, automobile, biomedicine, and electric cables insulation, including power cables in nuclear power plants (NPP) [1,2,3,4]. During normal operation, degradation takes place through internal or external processes which end up limiting the lifetime of the material. A crucial aging mechanism of polyethylene, as well as of many polymers, is oxidation; this process can be initiated by electronic excitations produced by irradiation (UV, γ-rays, electrons, swift heavy ions) [5,6,7]. However, due to the complexity of the oxidation mechanisms, many studies rely on the assumptions usually made for kinetic modelling of PE degradation [8,9,10,11,12,13,14,15]. For example, Niki et al. [16], in the first of a series of three papers, verified the fraction of the oxidation products by thermal decomposition based on their kinetic models in bulk atactic polypropylene. Later, other works were devoted to the quantification of the products under thermo- and photo-oxidation conditions by means of IR spectroscopy [17,18,19,20]. In particular, carbonyl groups (namely ketones) were found to be the majority products under thermo-oxidation conditions. Despite the fact that ketones can undergo Norrish-type reactions under photochemical conditions [21], they still are majority products [17]. The explanation of the kinetic path towards the formation of ketones and other carbonyl species have sparked a number of studies based on rate theory models essentially assuming homogeneous concentration (i.e., homogenous chemical kinetics) [15,22,23,24,25,26,27]; in these studies, important intermediate species include hydroperoxides (POOH groups). In a recent kinetic study by Da Cruz et al. [20], a new formation mechanism of ketones starting from two POOH is suggested when the bimolecular decomposition of POOH is the main source of radicals.

In the last decade, the idea of investigating the elementary mechanisms of PE degradation using ab initio calculations has emerged. Although kinetic constants and activation energies have been generally deduced from experiments by indirectly measuring the concentration of the products and assuming Arrhenius behaviour, there are reactions and processes which are difficult to probe experimentally such as H diffusion, abstraction, or parameters like transition ring size; the corresponding (free) energy barriers can be predicted by first principles calculations [28,29,30,31,32,33]. Especially, calculations have focused on the activation energy of reactions, because it crucially controls kinetic rate constants. However, describing PE under realistic conditions from first principles is a difficult endeavour, because of a complex microstructure which cannot be faithfully described by models whose size is limited to few tenths of atoms. Most studies focused on single molecular units in gas phase and the search of associated transition states; other atomic environments such as crystalline/amorphous lamellæ of polyolefins and their interfaces have not, or rarely [34,35], been tackled with ab initio methods. In particular, reaction processes in crystalline PE have been rarely considered, even though carbonyl defects and other species obviously affect the optical and electrical properties by forming shallow and deep traps [34,35,36,37]. Besides, the formation of defects in PE during radio-oxidation occurs due to the relatively low energy barrier for oxygen permeability, which is of the order of 0.4 eV [38], diffusing from the surface all the way through the amorphous regions; although it is generally assumed that crystalline regions are impermeable to oxygen at room temperature, [39] the relationships between crystallinity, density, and oxygen permeation at various conditions are not fully understood [38]. Not only the permeability plays a role, but also the pristine concentration of carbonyl groups does, which in commercial PE applications can reach 0.1%, resulting from the presence of oxygen impurities during polymerization [36]. It is therefore necessary to advance the atomic scale understanding of relevant chemical reactions through various models approaching crystalline and amorphous regions and to compare them to results for gas phase molecules. In this work, guided by the kinetic scheme focusing on the production of ketones through the intermediate hydroperoxide species, we investigate the activation energies by calculating full reaction paths using the climbing image nudged elastic band (CI-NEB) [40] method.

We discuss three main reaction pathways:

(i) The capture of oxygen by an alkyl radical;
(ii) The formation of hydroperoxides;
(iii) The decomposition of the latter.

Two models are considered: small molecules and crystalline PE. Although relevant reactions are supposed to occur mostly in the amorphous region, bimolecular reactions barriers calculated in the crystal can, in many cases, be safely transposed to the amorphous, as we will show. Reactions involving a hydroxyl radical are found to be particularly relevant for the decomposition of hydroperoxides and occur spontaneously regardless of the position of hydroxyl radical, at variance with reactions where hydroxyls are not present. Finally, we conclude that this radical is the best candidate leading to alkyl radical chain oxidation and the degradation of PE thanks to its reactivity.

## 2. Materials and Methods

All the results presented in this paper are based on density functional theory (DFT). Equilibrium structures and their total energies are obtained from the Quantum-Espresso software package (versions 6.4.1, 6.5 and 6.7) by using the PWSCF module [41]. The energy barriers and reaction pathways are calculated by the climbing image nudged elastic band (CI-NEB) method, as implemented in the Quantum-Espresso distribution. Quantum-Espresso is publicly available through the official site (http://quantumespresso.org (accessed on 21 June 2021)).

We rely on two models, the first one based on isolated molecules of varying length, the second one is crystalline PE, in the orthorhombic structure, as described in Ref. [37]. We will call them in the following molecular and solid models, respectively. While reactions occurring on isolated molecules might well represent unimolecular reactions occurring in low density regions of the amorphous, the crystal is the only one able to describe intermolecular (namely bimolecular) reactions. In some specific cases we will show the influence of density on reaction barriers, to assess the distribution of reaction energies due to local density variations.

**Figure 1:**
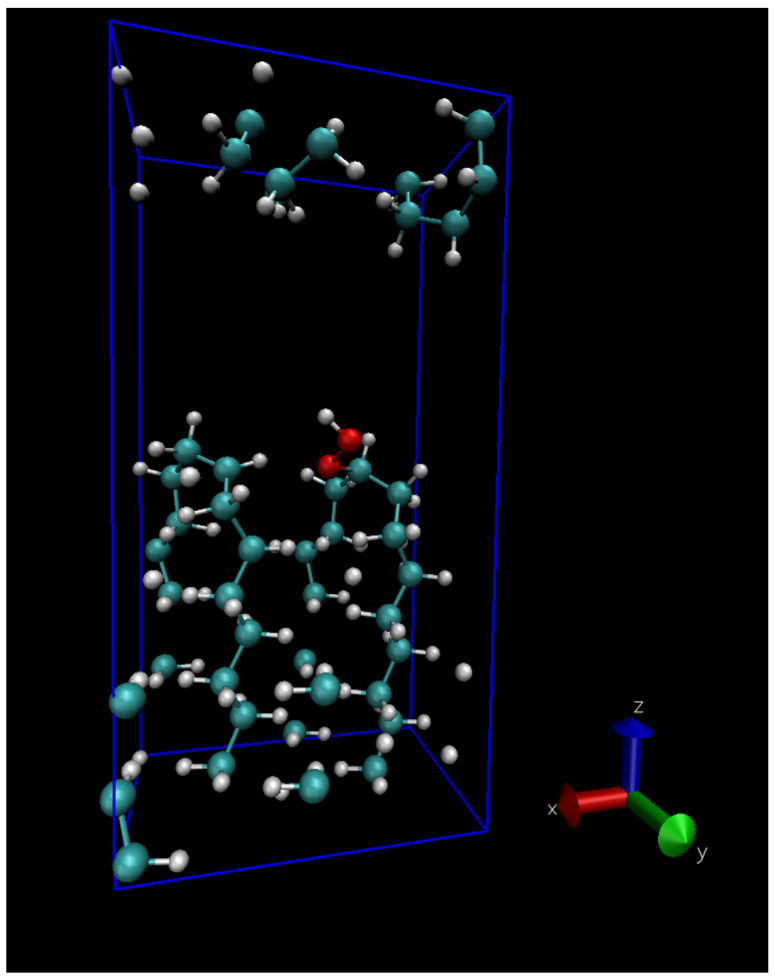
The periodically repeated 133-atom unit cell used to mimick the interface between two crystalline lamellæ of PE, here with a hydroperoxide.

For a few reactions we calculated the migration barrier in a different model mimicking the interface between two crystalline regions, with bent polymer chains at the crystal surfaces; the two facing surfaces are separated by approximately 5 Å. The unit cell of the lamellar model, containing 132 atoms for pure PE, is shown in Figure 1, similar to a model previously used to describe a carboxyl group grafted onto a lamella [34].

Pseudopotentials, norm-conserving (nc) for C and H and both nc and ultrasoft for oxygen, were generated as described in [37] and used with the optB86b+vdW exchange-correlation (xc) functional [42] and, in some cases, with the hybrid functional vdW-DF-cx0 [43].

The functional optB86b+vdW includes a gradient corrected short range xc contribution and a long-range non-local van der Waals correlation term in order to provide a good description of van der Waals interchain interactions in crystalline PE. In addition, for the accuracy of some specific reactions, we used the hybrid functional vdW-DF-cx0 which mixes into the same exchange part, a portion of Hartree–Fock exchange energy to improve the description of electronic density localization. For molecular models, we used body-centred tetragonal unit cells in order to maximise the chain ends distance between periodic images of the molecules; the unit cells were 40 × 40 bohr wide in the plane perpendicular to the chain and they exceeded the molecules length in the direction parallel to the chain so to have at least a distance of 25 bohr between atoms of two different periodic images. The unit cell of the crystalline solid is orthorhombic, containing 12 atoms, and we sampled the BZ with a 3 × 3 × 6 Γ-centred regular **k**-point mesh. To model isolated defects in the solid we used a 2 × 1 × 4 supercell (containing 96 atoms plus defects), and we employed a 2 × 3 × 2 Γ-centred **k**-point mesh. The theoretical equilibrium lattice parameters of the orthorhombic unit cell in atomic units were: a = 9.18 Bohr, b = 13.14 Bohr, and c = 4.84 Bohr, and for the 2 × 1 × 4 supercell: a = 18.36 Bohr, b = 13.14 Bohr, and c = 19.36 Bohr. As our NEB calculations revealed a quite low migration barrier for the oxygen molecule in the crystal, we performed a Car–Parrinello molecular dynamics simulation in the NVT ensemble using the cp.x executable in the Quantum-Espresso distribution. The simulation was run for 4.6 ps with a time step of 0.12 fs and a fictitious electronic mass of 300 atomic units.

## 3. Results

### 3.1. Oxygen Capture by an Alkyl Radical

**Figure 2:**
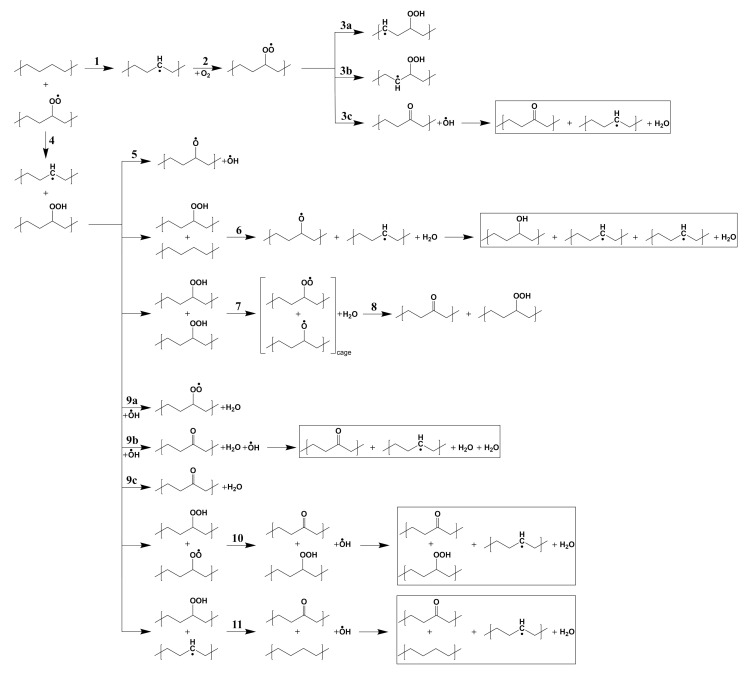
A summary of reaction paths leading to the (radio)-oxidation of polyethylene in the form of ketones. Starting from the production of alkyl radicals (typically by irradiation, reaction 1), the capture of O${}_{2}$ molecules follows (reaction 2). The chain reaction can proceed through the formation (then decomposition) of hydroperoxides either by unimolecular reactions 3a–c, or by bimolecular reaction 4 and then unimomolecular or bimolecular reactions 5–11. The main stable final products are ketones and water molecules, while hydroperoxides, hydroxyl radicals and peroxy radicals will take part in further reactions, together with alkyl and alkoxy radicals.

Building on previous works [20,31] we present in Figure 2 a summary of most probable reaction pathways leading to the oxidation of polyethylene. We assume that alkyl radicals exist through the C-H bond dissociation by γ-irradiation (reaction 1).

**Figure 3:**
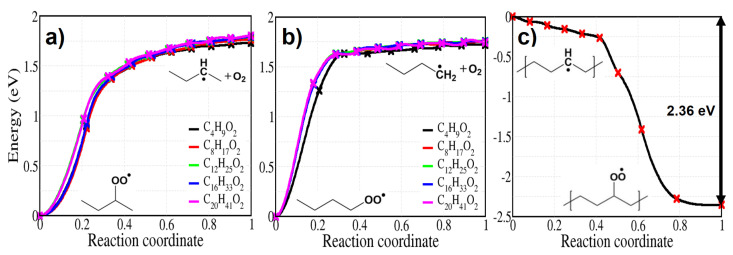
The capture of an oxygen molecule by an alkyl radical occurs spontaneously, as shown by the energy profiles shown here: (**a**) capture by an alkyl radical situated on a internal carbon on alkane molecules of varying length (**b**) capture by an alkyl radical situated on an end-chain carbon of alkane molecules of varying length (**c**) capture by an alkyl radical in a crystalline region of PE.

The reported C-H bond energies from methane to propane are in the range of 407.6–439.7 kJ/mol (4.25–4.58 eV) from experiments and 397.5–437.6 kJ/mol (4.14–4.56 eV) from calculations [30,44]. After the formation of an alkyl radical, an oxygen molecule can be captured without any energy barrier at a –${}^{•}$CH– site by forming a peroxy radical (reaction 2). Figure 3 presents the energy profiles of reaction 2 for both the molecular and the solid models.

We checked the reaction for molecules of varying lengths and both for capture far from chain end (approximately in the middle of the molecule, Figure 3a) or at chain end (Figure 3b). Addition of O${}_{2}$ shows barrierless energy profile not only for the various length of alkyl chains but also for crystalline PE, with high exothermic enthalpies of 1.78 eV (chain centre, average), 1.75 eV (chain end, average), and 2.36 eV (PE crystal), which imply spontaneous $O_{2}$ capture by an alkyl radical.

**Figure 4:**
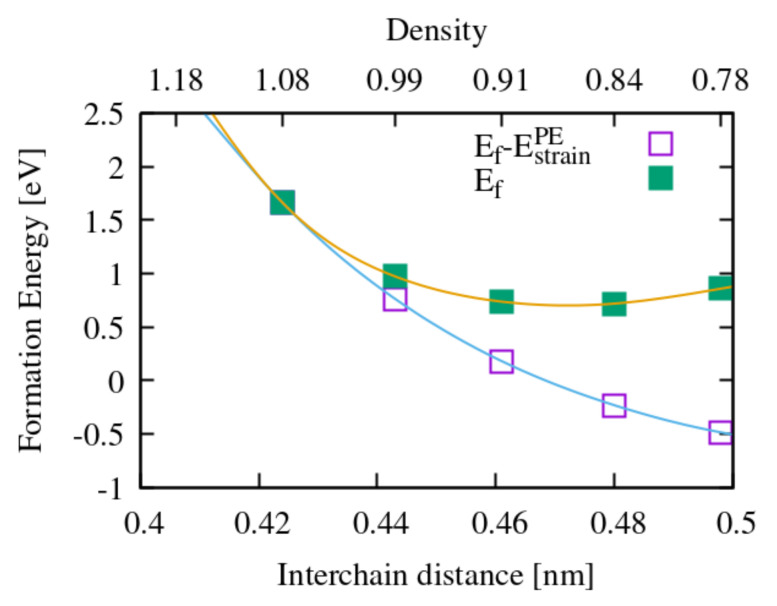
Solution energy of an oxygen molecule in crystalline PE as a function of the interchain distance. For both the yellow and the blue curves the oxygen molecule is inserted in a model of PE with scaled in plane lattice parameters, so to modify the interchain distance and not the dimensions along the chain. The equilibrium interchain distance is 0.424 nm; the yellow curve gives the standard solution energy (E${}_{f}$), computed with respect to the equilibrium structure; the blue curve takes as a reference a PE crystal with the same scaled lattice parameters as that of the supercell hosting the molecule, that is we remove the strain energy required to dilate the PE at 0 K (E${}_{strain}^{PE}$). The oxygen chemical potential, in both cases, is the energy of an isolated O${}_{2}$ molecule.

In crystalline PE, at variance with gas phase reactions, the diffusion of oxygen represents a limiting step. Considering the complex microstructure of PE, with crystalline and amorphous regions and interfaces between them, a full account of energy barriers for oxygen diffusion will be a study in itself. However, useful hints emerge from our calculations of a molecule in solution into crystalline PE. First, the molecule does not spontaneously dissociate. Second, the solution energy of the molecule (calculated with respect to pristine crystalline PE and a gas phase oxygen molecule) is relatively high at constant volume, but varies considerably when varying the interchain distance of PE. This can be appreciated from Figure 4, which suggests a behaviour similar to O${}_{2}$ in amorphous silica [45]: diffusion takes place in the amorphous more easily than in the crystal, thanks to a wide distribution of interchain spacings; this view corroborates the usual assumption that oxygen in PE essentially diffuses through the amorphous regions [46].

The typical activation energies associated to oxygen dissolving and diffusing in PE range from 0.35 to 0.45 eV [46]. However, in this case, this activation energy might be actually related to permeation instead of diffusion, because, at variance with SiO${}_{2}$ where bottlenecks exist between the voids where the molecule can easily sit, in PE the channels between alkyl chains constitute an easy diffusion path. To check this we performed a NEB calculation for the migration of an oxygen molecule between two equivalent neighbouring insertion sites along the channels and found a migration barrier on the order of 0.1 eV. To corroborate this results we also performed an ab initio molecular dynamics simulation which, although not long enough to provide an accurate value of the free energy barrier, featured a number of jumps of the molecule consistent with the migration barrier calculated with the NEB method. This result, combined with those in Figure 4, suggests that the previously cited permeation activation energy (0.4 eV [38]) stems essentially from thermodynamics (i.e., the solution energy) and not from kinetic barriers, and probably represents an average of the solution energy over the available insertion sites, mainly in the amorphous and in the interface regions. In real samples we can imagine a variety of reasons why the concentration of O${}_{2}$ molecules, in both the amorphous and the crystalline regions, is not the equilibrium one: quenching from higher temperatures, recrystallization during irradiation, extrusion or material synthesis under higher oxygen pressure. For these reasons, the presence of O${}_{2}$ cannot be categorically excluded in the crystalline regions.

### 3.2. Formation of Hydroperoxides

After the capture of O${}_{2}$ by an alkyl radical (reaction 2), the reaction pathway branches into several possible channels towards the formation of hydroperoxide groups. Let us first consider the formation of a hydroperoxide by H-abstraction from the same alkyl chain; depending on its positon it is labeled γ, β, or α (reaction 3a, 3b, and 3c). Reaction 3c does not form the hydroperoxide but a ketone and a water molecule, because its intermediate configuration, which is α-alkyl-hydroperoxy radical, dissociates in less than 20 μs [47,48]. We do not consider H abstraction from more distant sites (e.g., from δ-position) because the alkyl chain is hardly bent in crystalline PE, which would presumably lead to unfavourable pathways.

**Figure 5:**
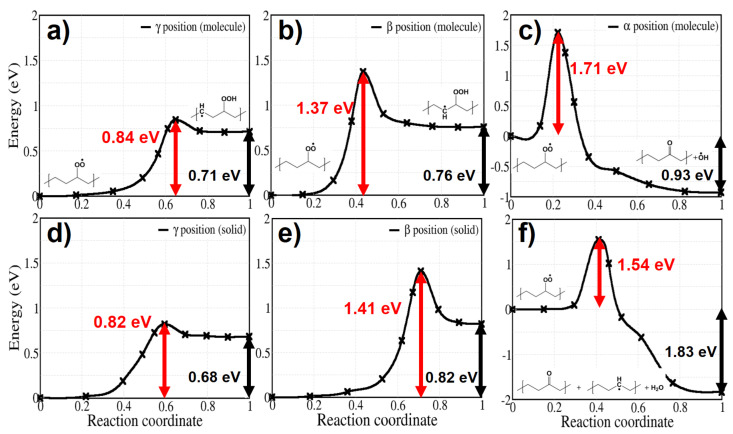
Energy profiles for reactions labeled 3a–c in Figure 2. Panels (**a**–**c**): H-abstractions from γ,β,α, respectively, in a molecular model. Panels (**d**–**f**): analogous reactions for crystalline PE.

Figure 5 shows calculated activation energies for both molecular and solid models. The activation energy of H abstraction from the γ position has the lowest values among the H abstractions: it amounts to 0.84 eV and 0.82 eV for the molecular and the solid model, respectively, (Figure 5a,d). As a hint on the influence of density on the energy barriers in solid PE, we calculated the barrier of reaction 3a at several interchain distances, from 0.42 to 0.48 nm; the variation of the energy barrier does not exceed 0.05 eV.

**Figure 6:**
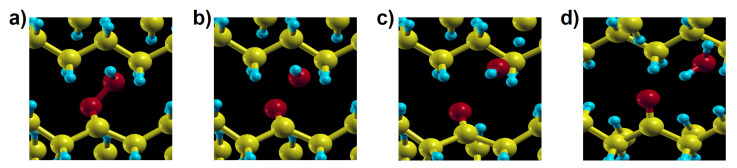
The four snapshots (**a**–**d**) are representative steps of the relaxation from an (unstable) α-alkyl-hydroperoxy radical to the endpoint of reaction 3c in crystalline environment.

H abstraction from β position shows higher energy barriers of 1.37 eV and 1.41 eV for the molecular and the solid model, respectively (Figure 5b,e). γ and β hydrogen abstraction both are endothermic, which means that a reverse reaction is more likely than the forward one, reducing the effective rate constant of hydroperoxide production. In contrast, α hydrogen abstraction is exothermic, with the final equilibrium structure having much more stable energy than for the other two mentioned reactions. Although this reaction would directly lead to ketone products as observed in thermo- or photo-oxidation of PE, it has a high activation energy (1.71 eV for the molecular model and 1.54 eV for the crystal) and is thus unlikely to occur in normal conditions. In contrast with reaction 3a and 3b, showing similar behaviour in the solid and for the gas phase molecules, reaction 3c in crystalline PE leads to a different final product: during the reaction a hydroxyl radical is produced from P${}^{•}$O–OH dissociation and reacts with another neighbouring polymer chain, forming an alkyl radical and a water molecule in crystalline PE. This additional process occurs spontaneously, as seen from the structural relaxation in Figure 6. Although the instability of α-alkyl-hydroperoxy radical and the reactivity of hydroxyl radical have already been investigated [47,48,49] in simple molecular systems, not much has been done concerning these reactions in crystalline polymers nor their role in a global kinetic pathway. Regarding the hydroxyl radical, its role in catalysing hydroperoxide formation will be discussed in detail with reaction 9. Reactions involving additional steps are marked by rectangles in Figure 2.

**Figure 7:**
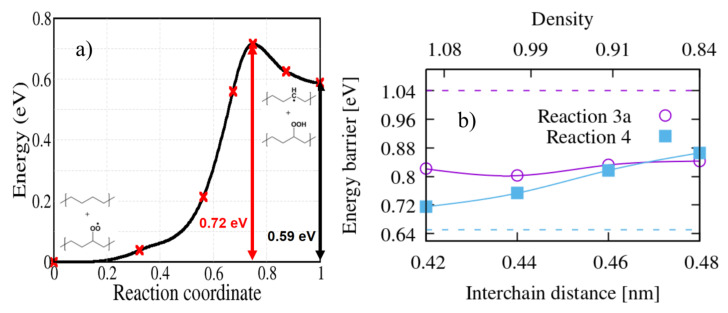
(**a**) The energy profile calculated for the bimolecular reaction 4 of Figure 2 as calculated in crystalline PE. (**b**) The influence of the PE interchain distance on the energy barriers of bimolecular reactions 3a and 4. The dashed lines are the energy barriers calculated for the same reactions in the lamellar interface structure.

On the other hand, hydroperoxides can be formed also when the peroxy radical grabs an H atom from an adjacent polymer chain in the system. In order to simulate this intermolecular abstraction we chose the H atom which is closest to the peroxy radical, in crystalline PE (reaction 4); for all bimolecular reactions we limited our study to the crystalline system. The calculated activation energy is 0.72 eV (Figure 7). This reaction is competitive with γ-intramolecular H abstraction (Figure 5a,d) because they both contribute to the formation of a hydroperoxide. In previous kinetic modelling, this sort of reaction was treated with an activation energy of 0.76 eV [15,22], not far from our calculated value.

The energy barrier of intermolecular reaction 4 depends more strongly on the interchain distance than the intramolecular reaction 3a: the value of 0.72 eV corresponds to the zero temperature theoretical equilibrium structure (interchain distance 0.42 nm), while if we stretch the interchain distance to the value of 0.46 nm (corresponding approximately to the room temperature density of LDPE) the energy barrier is raised to 0.82 eV. This gives a hint on the distribution of energy barrier for hydroperoxide formation by intermolecular H-abstraction in the amorphous region, where interchain distance can locally vary. Apart from density, however, the local conformation of the chains can also play a role; for the sake of illustration we computed the energy barrier of reactions 3a and 4 using the lamellar model shown in Figure 1. The resulting energy barriers (see dashed lines on Figure 7b) seems to point to the fact that bent chains substantially raise the barrier for the unimolecular reaction 3a, while lower slightly the energy barrier of the bimolecular reaction 4; of course this will somewhat depend on the choice of the interface site where the peroxy radical is grafted, in our case the final state of Reaction 4 (the hydroperoxide) is shown in Figure 1.

### 3.3. Decomposition of Hydroperoxides

**Figure 8:**
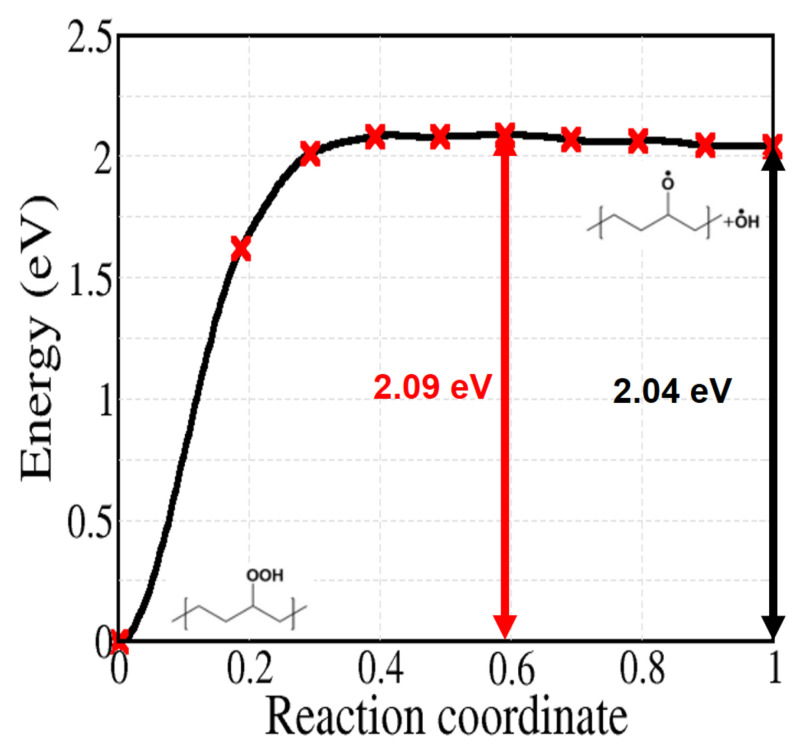
Calculated energy profile for reaction 5 of Figure 2.

**Figure 9:**
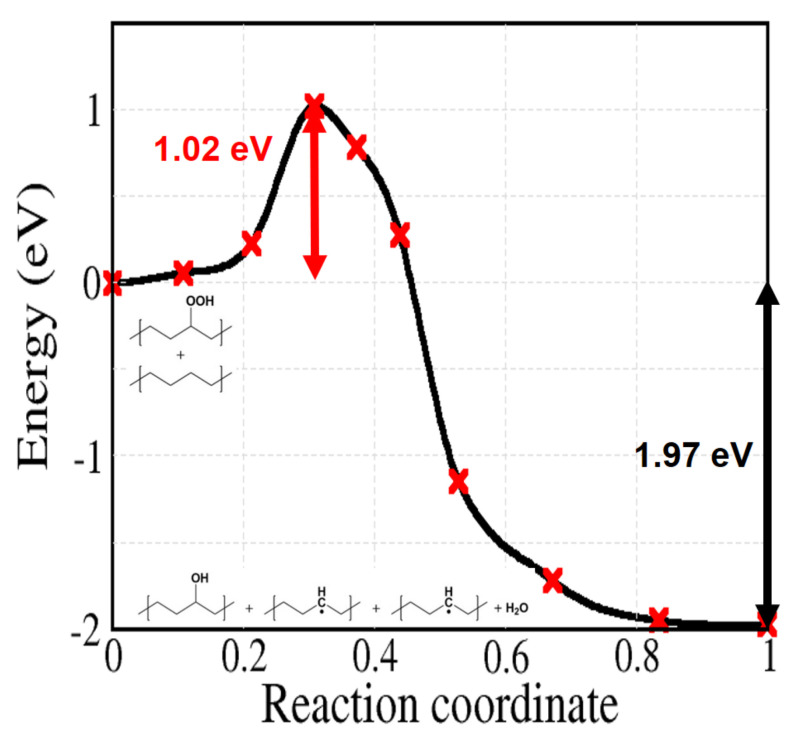
Calculated energy profile for reaction 6 of Figure 2 in a model of crystalline PE. The calculated outcome is here different from the commonly accepted one, giving instead two alkyl radicals and an alcohol, plus a water molecule.

**Figure 10:**
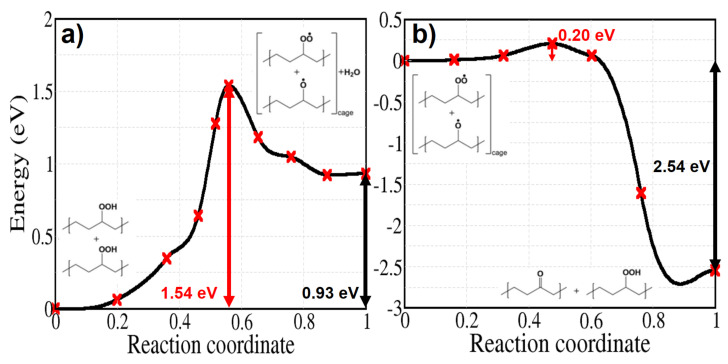
Energy profiles for reactions 7 (**a**) and 8 (**b**) of Figure 2 calculated for crystalline PE.

Reaction 5 to 9 of Figure 2 are related to the decomposition of hydroperoxides, which can occur both by unimolecular or bimolecular processes. At first, we can simply consider the unimolecular PO–OH bond thermal dissociation (reaction 5). As shown in Figure 8, this reaction shows the highest activation energy (2.09 eV) among the reactions in Figure 2. From the experimental kinetics of hydroperoxide decomposition, the contribution of this reaction is only up to 2% for temperatures below 200 ${}^{\circ }$C, indicating the importance of pseudo-unimolecular POOH decomposition such as reaction 6 when hydroperoxide concentration is low ([PH] >> [POOH]) [33,48,50]. When the concentration of POOH is small enough, reaction 6 (Figure 9) would also prevail over reaction 7 (Figure 10a). However, for the former reaction, the traditionally accepted reaction pathway shown in Figure 2 comes into question. In fact, according to our calculations, it proceeds to a different product: the alkoxy radical in crystalline PE spontaneously abstracts an H atom from another alkyl chain, forming an alcohol as shown in Figure 9 (this further, unexpected, step is shown in the box after reaction 6 in Figure 2).

The obtained activation energy is 1.02 eV. This reaction, among the considered ones, is the one that forms the most alkyl radical chains with comparatively low activation energy. These alkyl radicals can diffuse along or across the chain. Otherwise, with the presence of oxygen, alkyl radicals do not propagate and grab oxygen as in reaction 2 by forming peroxy radical defects.

**Figure 11:**
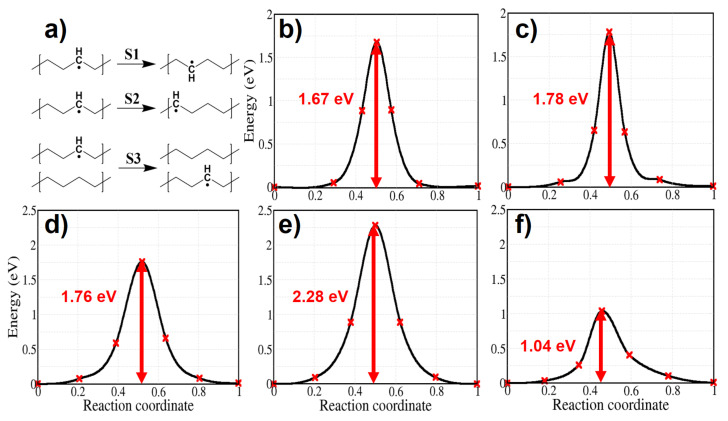
Energy profiles for the migration of an alkyl radical. Panel (**a**): schemes for nearest neighbor (S1) and second nearest neighbor (S2) migration along the alkane chain, and from a chain to a neighboring one (or across the chains, S3). Panels (**b**,**c**): energy profiles for migrations S1–S2 for the molecular model. Panels (**d**,**e**): migrations along the chain for the solid model; panel (**f**): migration across the chains (S3) in crystalline PE.

We calculated the activation energy for the migration of an alkyl radical through three possible atomic jumps and we show it in Figure 11. Shimada et al. [51,52] compared the decay rate of alkyl radicals trapped in the urea-polyethylene complex and in solution grown crystals. Although the mobility of PE chains is higher in the urea-polyethylene complex, alkyl radicals decay at slower rate here than in the solution grown crystals, where interchain motion is reduced. This finding implies that the rate of alkyl radical propagation across the chains is much faster than that along the chain in polyethylene crystals. In agreement with such a result we find an activation energy for alkyl radical migration from chain to chain of 1.04 eV, which is much lower than the activation energy for alkyl migration along the chain both in the molecular and the solid models (see Figure 11).

The bimolecular decomposition of POOH (reaction 7) is relevant when the concentration of hydroperoxides is sufficiently high or when an inhomogeneous distribution of hydroperoxides leads to local accumulations of these groups and may become the main free radical formation reaction [53]. Figure 10a shows the energy profile of reaction 7, which has a relatively high energy barrier of 1.54 eV. After the hydroperoxide decomposition the reaction takes place depending on the existence of the so called cage effect—i.e., the proximity of the two radicals—and their tendency to diffuse away from each other along the alkyl chains. When the radicals are trapped in the cage without diffusing, the reaction forms a hydroperoxide and a ketone by abstracting the tertiary H atom of the alkoxy radical. While this process has been supposed to happen with no activation energy, our calculation shows a small activation energy of 0.20 eV (Figure 10b) [54,55].

Apart from the previous reaction, a hydrogen from a secondary hydroperoxide can be attacked by other radical species resulting from the products of other PE oxidation reactions. We considered three types of radicals: (i) a hydroxyl (ii) a peroxy radical, and (iii) an alkyl radical. The hydroxyl radical has a high reactivity and easily abstracts a H atom from the alkane. De Saint-Claire [33] reported a rate constant (10${}^{12-13}$ cm${}^{3}$ mol${}^{-1}$ s${}^{-1}$) of H atom abstraction by hydroxyl radical, which is much higher than that of other reactions, but coherent with previous experimental estimations for ${}^{•}$OH radical reactions with alkanes [56,57]. The induced decomposition by the radicals amounts up to 54% of the overall hydroperoxide decomposition channel in thermo-oxidative conditions. Reactions with hydroxyl radical in crystalline PE are essential because the radical can exist not only from products of reactions but also from outside of crystalline PE, namely the amorphous phase. Moreover, the permeability of PE to the hydroxyl radical is expected to be comparatively higher than to oxygen [39], because the ${}^{•}$OH radical size is smaller than O${}_{2}$. Therefore, we tested these reactions by putting a hydroxyl radical next to a hydroperoxide on primary and tertiary sites for reaction 9a and 9b, respectively. Reaction 9a produces a peroxy radical and a water molecule. For reaction 9b, firstly, the hydroxyl radical abstracts the tertiary H atom of the hydroperoxide. As an intermediate state an α-alkyl-hydroperoxy radical and a water molecule are formed, then the P${}^{•}$O–OH bond is decomposed immediately by forming a ketone and a hydroxyl radical. Both these reactions occur spontaneously. In order to check our initial position we performed relaxations constraining the distance between the tertiary H atom of the secondary hydroperoxide and the oxygen atom of the hydroxyl radical at various intermediate values. As for the oxygen capture from an alkyl radical, our calculations show that no barrier needs to be overcome to trigger this reaction.

We also verified by hybrid functional calculations the energy profile of those relaxations, confirming the spontaneous H-abstraction by the ${}^{•}$OH radical.

**Figure 12:**
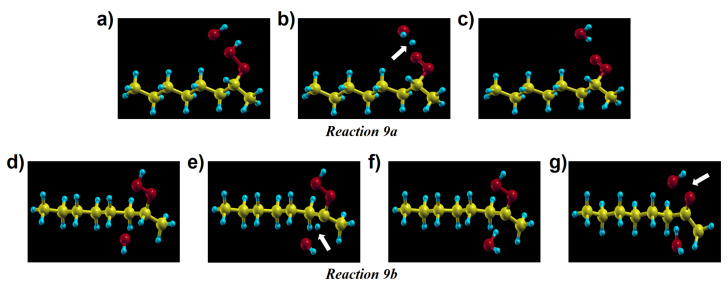
Intermediate steps of reactions 9a (panels (**a**–**c**)) and 9b (panels (**d**–**g**)) of Figure 2, showing two possible channels of hydroperoxide decomposition due to the action of a hydroxyl radical in a molecular model. The different outcome of the two reactions stems from the initial position of the hydroxyl relative to the hydroperoxide group.

**Figure 13:**
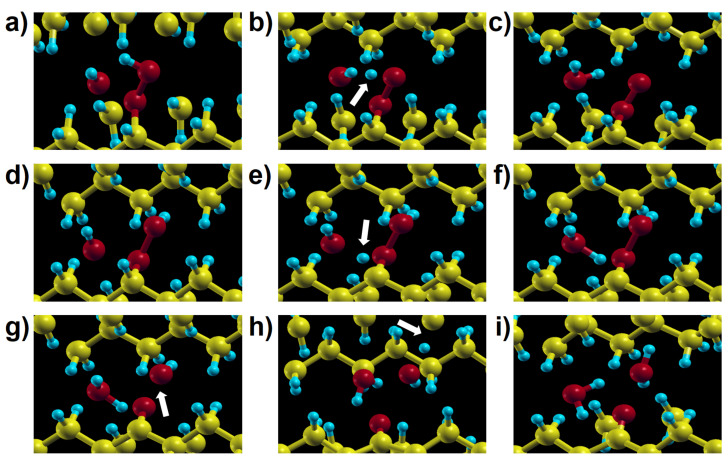
Intermediate steps of reactions 9a (panels (**a**–**c**)) and 9b (panels (**d**–**i**)) in Figure 2 in crystalline PE. The outcome of reaction 9b in the solid is different from the analogous one in the molecular model (Figure 12d–g), because here the remaining ${}^{•}$OH radical reacts with a neighbouring chain (see the arrow in panel (**h**)) giving one more water molecule (panel (**i**)).

Figure 12a–g shows intermediate steps along the structural relaxations corresponding to reactions 9a and 9b, respectively. A hydroxyl radical abstracts a H atom spontaneously, without any barrier. In particular, we could see that a new hydroxyl radical is again present in the final state of reaction 9b; this ${}^{•}$OH radical can react with other molecules or alkyl chains successively. This is important because, as we confirmed dealing with reaction 3c in crystalline PE, hydroxyl radical can be available for these reactions to proceed spontaneously. Intermediate steps of the structural relaxation corresponding to reaction 9a and 9b in crystalline PE are presented in Figure 13. While reaction 9a in Figure 13a–c shows the same products as its molecular model in Figure 12a–c, the decomposed hydroxyl radical from the hydroperoxide in reaction 9b abstracts a H atom from another alkyl chain producing an alkyl radical chain (Figure 13d–i). The overall reaction proceeds as follows:

(1) $$POOH+{}^{•}OH\to P^{•}OOH+H_{2}O\to P=O+P^{•}+2H_{2}O.$$

Note that reactions 9a and 9b, as well as 3c, could occur also in amorphous regions, similarly to the crystal, whenever two polymer chains are close enough.

**Figure 14:**
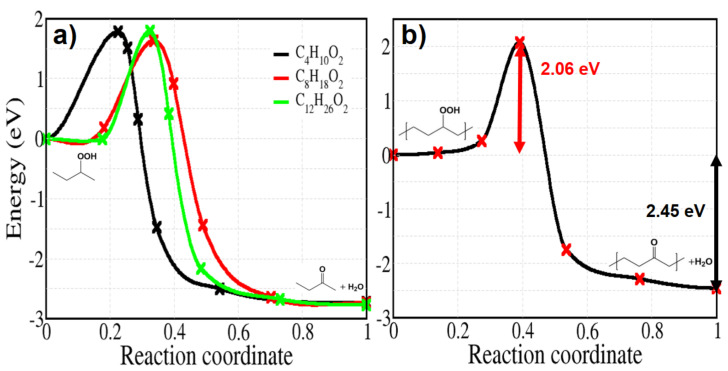
Energy profiles calculated for reaction 9c of Figure 2. Panel (**a**): molecular models of varying sizes. Panel (**b**): crystalline PE.

Finally, we deal with reaction 9c of Figure 2, starting from a hydroperoxide without hydroxyl radical and giving a ketone and water in the final state; the corresponding activation energy is calculated by varying the number of carbon from 4 to 12 for comparison (Figure 14a). In contrast with the previous reactions 9a and 9b of spontaneous H abstraction, the barriers here are quite large, regardless the size of the molecules, showing an average value of 1.73 eV. The activation energy for the solid model also has the high value of 2.06 eV (Figure 14b). The energy is not far from that of reaction 5, describing the dissociation of a PO–OH bond.

**Figure 15:**
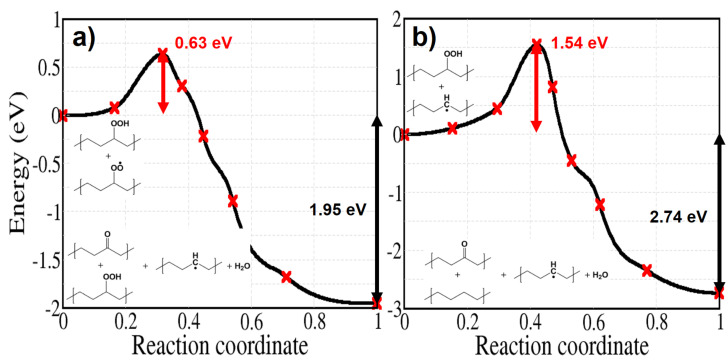
Energy profiles for hydroperoxide decomposition reactions induced by (**a**) a peroxy radical (**b**) an alkyl radical, sitting on a nearby polymer chain.

Energy profiles of free radical induced decomposition of hydroperoxides are shown in Figure 15. They both attack a tertiary hydrogen atom of a secondary hydroperoxide in the same manner as in reaction 9b. Although the activation energy of reaction 10 is higher than that for hydrogen abstraction by hydroxyl radicals, we can observe that a hydroperoxide is regenerated during the reaction; this is important during the initial stages of the oxidation [58,59], when the formation of ketones proceeds at a constant rate. In contrast with reaction 9b and 10, the activation energy of reaction 11 is high and amounts to 1.54 eV. This implies that hydrogen abstraction at a tertiary site is not seriously affected by an alkyl radical sitting on a nearby polymer chain.

If we consider the decomposition of hydroperoxides in absence of radical attacks, the easiest path is through reaction 6, whose activation energy is 1.02 eV, all others having higher activation energies. In contrast, a hydroxyl radical spontaneously removes the H atom of hydroperoxides, as well as other H atoms in alkyl chains. In other words, all H abstractions related to hydroxyl radical (reaction 3c, 9a, and 9b) showed barrierless energy profiles. Especially during reaction 9b, a new hydroxyl radical is again generated after the abstraction of tertiary hydrogen, potentially triggering a chain reaction with other alkyl radicals. Besides, a hydrogen abstraction by a peroxy radical also involves an energy barrier which is low compared to other hydroperoxide decomposition reactions, by forming an additional hydroperoxide as a product. Therefore, the role of peroxy and, even much more, hydroxyl radicals, causing chain reactions, cannot be overlooked for the formation of ketones or other radical species and seems to be crucial for the oxidative degradation PE.

## 4. Discussion

As a basis for discussion all calculated activation energies are tabulated in Table 1.

Assuming the production of alkyl radicals, and the spontaneous capture of oxygen molecules by the latter, as shown in Section 3.1, the next important step is the formation of hydroperoxides. The reactions showing the lowest barriers for this process are reactions 3a and 4. However, the reverse barrier is small (only a few tenths of an eV), lower than the forward barrier and of the diffusion barrier for the alkyl radical, suggesting that the effective rate of these reactions is low, because the hydroperoxide can easily decompose in its constituents, peroxy radical and a restored alkyl chain. Another possible direct channel of ketone formation, without hydroperoxide intermediates, could possibly stem directly from reaction 8 of Figure 2, provided that alkoxy radicals are available and get close to peroxy ones. This might be facilitated by the reaction of hydrogen molecules with peroxy radicals.

Apart from the reverse reactions of 3a and 4, hydroperoxides can decompose following reaction 6 (barrier 1.02), possibly followed by an oxygen capture by the alkyl and a subsequent reaction 8 (barrier 0.2 eV). The highest barriers involved in those processes are not very easy to overcome at room temperature, although for reactions 3a and 4 the rate might be enhanced by a sufficiently large concentration of alkyl radicals. Hydroperoxide decomposition might be enhanced by the presence of hydroxyl radicals, however their production through reactions 3c and 5 is hindered by a high energy barrier (>1.7 eV).

The relative importance of the various formation and decomposition reactions of hydroperoxides depends thus on their initial concentration and that of various types of radicals. A direct experimental probe of the concentration of the various species, including in particular hydroperoxides, would be of great help to further unravel the degradation mechanisms in polyethylene.

## 5. Summary and Conclusions

Degradation mechanisms of oxidation PE were investigated by calculating reaction barriers in small molecules and crystalline polyethylene. We divided our study in three main parts: (i) oxygen capture by an alkyl radical, (ii) formation of hydroperoxides, and (iii) decomposition of hydroperoxides. First, oxygen reacts with alkyl radicals spontaneously, even though diffusion through crystalline PE might constitute a limiting step.

After the formation of peroxy radicals, hydroperoxides are formed by intramolecular or intermolecular H abstractions. We considered the H abstraction from α, β, and γ positions. Among the intramolecular reactions, abstraction from the γ position has the lowest activation energies, which are 0.84 eV and 0.82 eV for molecular and solid model, respectively. The energy barrier of intermolecular H abstraction is 0.72 eV. Therefore, competition between these two reaction pathways is probable in crystalline PE.

Although, as we show, the solubility of oxygen in crystalline regions is expected to be much lower than in the amorphous, for many bimolecular reactions the energy barriers in the crystal should provide a reasonable estimate for the amorphous regions, as we have shown by varying the interchain distance.

In contrast with the typical assumptions made in the literature, our calculations show that some reactions such as reaction 3c (α–H abstraction), 5–6 (PO–OH bond cleavage), and 9b (POOH decomposition through ${}^{•}$OH radical), have different outcomes whether the reaction occurs in crystalline (and probably also amorphous) PE or in an isolated alkane molecule. In particular, in presence of hydroxyl radical, further reactions take place and the activation energy of decomposition of hydroperoxide varies largely. Without the radical, the reaction of bimolecular POOH decomposition leading to an alcohol, an alkyl radical and a water molecule has the minimum activation energy of 1.02 eV. For isolated molecules, the activation energy of the unimolecular dissociation of PO–OH is much higher, 2.09 eV. In contrast, H abstraction by hydroxyl radical is spontaneous regardless of its position, both in the crystal and on an alkane molecule. Especially, H abstraction from a hydroperoxide in tertiary site leads to successive reactions with other alkyl chains and finally leaves an alkyl radical chain. Comparing the energy of C-H bond dissociation in pure crystalline PE, which is up to 439.7 kJ/mol, this mechanism forming alkyl radical chain is much more favourable. Therefore, we conclude that, even in presence of small concentrations of hydroxyl radicals, the reactions they induce may lead to a critical degradation of crystalline PE.

We believe that our study of activation energies and reaction processes provides information at the atomic scale which can be hardly obtained by experiments, and gives insights on polymer degradation mechanism which are useful for further kinetic studies.

## Figures and Tables

**Table 1:** Activation energies calculated in the present paper for the reactions shown in Figure 2. Energies are in eV.

Reaction		Activation Energy [eV]	
Description	Label in Figure 2	Molecule	Crystal
oxygen capture	2	no barrier	no barrier
γ–H abstraction	3a	0.84	0.82
β–H abstraction	3b	1.37	1.41
α–H abstraction	3c	1.71	1.54
bimolecular H-abstraction	4	–	0.72
unimolecular PO–OH bond cleavage	5	2.09	–
pseudo-unimolec. POOH decomposition	6	–	1.02
bimolecular POOH disproportionation	7	–	1.54
bimolecular alkoxy/peroxy reaction	8	–	0.2
POOH decomposition by ${}^{•}$OH (1)	9a	no barrier	no barrier
POOH decomposition by ${}^{•}$OH (2)	9b	no barrier	no barrier
unimolecular POOH decomposition	9c	1.73 (average)	2.06
POOH decomposition by POO${}^{•}$	10	–	0.63
POOH decomposition by P${}^{•}$	11	–	1.54

## Data Availability

The data presented in this study are available on request from the corresponding author.
